# Computed tomography-based investigation of degenerative neural cervicothoracic foraminal stenosis as a potential mechanism for Horner syndrome

**DOI:** 10.3389/fopht.2024.1497845

**Published:** 2025-01-07

**Authors:** Joshua Ong, Mariko Kurokawa, Sangeeta Khanna, Lindsey B. De Lott, Ryo Kurokawa, Aseem Sharma

**Affiliations:** ^1^ Department of Ophthalmology and Visual Sciences, University of Michigan, Ann Arbor, MI, United States; ^2^ Divison of Neuroradiology, Department of Radiology, University of Michigan, Ann Arbor, MI, United States

**Keywords:** Horner syndrome, degenerative foraminal stenosis, computed tomography, computed tomography angiography, neural cervicothoracic foraminal stenosis

## Abstract

**Purpose:**

To investigate the presence and/or severity of cervicothoracic foraminal stenosis between the C7 and T3 segments could account for Horner syndrome, otherwise deemed to be idiopathic in nature.

**Methods:**

This study was an IRB-approved, retrospective study that included 28 patients [mean ± standard deviation (age: 54.5 ± 18.7 years)] with a confirmed diagnosis of Horner syndrome, absence of any identifiable cause, and availability of a neck CT/CT angiogram obtained within 6 months of the diagnosis. A neuroradiologist masked to the laterality of Horner syndrome reviewed the CT scans, documenting the presence/severity of foraminal stenosis at levels C7-T1, T1-2, and T2-3 on a 4-point Likert scale defined as follows: 0, none; 1, mild; 2, moderate; and 3, severe narrowing, with grades 1, 2, and 3 signifying <1/3rd, 1/3rd–2/3rd, and >2/3rd stenosis of the expected normal foraminal dimension.

**Results:**

Foraminal stenosis was present ipsilateral to the side of Horner syndrome in four (14.3%) patients and on the contralateral side in five (17.8%) patients. No significant difference in the extent of ipsilateral and contralateral foraminal stenosis was present at C7-T1 (*p* = 0.66), T1-2 (*p* = 0.32), or T2-3 (*p* = 0.75) levels. Mean ipsilateral (0.33 ± 1.0) and contralateral (0.33 ± 1.1) cumulative foraminal stenosis ddddscores were not significantly different (*p* = 1). Mean maximum foraminal stenosis scores ipsilateral (0.22 ± 0.59) and contralateral (0.30 ± 0.81) to the side of Horner syndrome were also comparable (*p* = 0.54).

**Conclusion:**

With the low prevalence of foraminal stenosis at C7-T3 segments and the equivalent prevalence and severity of foraminal stenosis ipsilateral and contralateral to the side of Horner syndrome, foraminal stenosis is unlikely to be a common causative mechanism for Horner syndrome.

## Introduction

Horner syndrome arises from the disruption of the oculo-sympathetic pathway, resulting in a classic triad of ptosis, miosis, and anhidrosis ipsilateral to the side of disruption (1, 2). The oculo-sympathetic pathway courses through various anatomical structures and consists of a three-neuron pathway starting in the hypothalamus. Sympathetic first order neurons from the hypothalamus descend, synapsing in the cervical spinal cord at the ciliospinal center of Budge located at cervicothoracic junction (1). Second-order neurons leave the spinal cord along the C8, T1, and T2 nerve roots, exiting the spinal canal through the C7-T3 neural foramina, and then course through the cervical sympathetic chain to synapse at C3/C4 within the superior cervical ganglion (1). Third-order neurons leave the superior cervical ganglion and course into the head through the internal carotid artery adventitia, eventually leading to the orbit (3). Neuroimaging is vital to identifying the underlying cause of disruption along the long course of the oculo-sympathetic pathway when the cause of Horner syndrome is not obvious.

Despite the frequent utilization of imaging, 23% to 80% of cases end up being classified as idiopathic (4–6). Case reports suggest that spinal degenerative changes may be an under-recognized cause of Horner syndrome. Observations of cervicothoracic spinal root cysts near the neural foramina at the cervicothoracic junction disrupting the second-order sympathetic neuron have been suggested as an underlying cause (7–9). However, a possible association of Horner syndrome with neural foraminal narrowing resulting from degenerative changes in the spinal column at the cervicothoracic junction has not been rigorously explored. Our aim was to investigate whether the presence and/or severity of foraminal stenosis between C7 and T3 segments could serve as an underlying etiology for Horner syndrome in such cases that were otherwise deemed idiopathic.

## Methods

This study was an IRB-approved, retrospective study that analyzed imaging and clinical data from patients aged 18 years or older with Horner syndrome over a 10-year period (2012–2022). We utilized our institutional search engine to identify patients with a diagnosis of Horner syndrome based on the ICD10 code G90.2. For this study, only patients with a diagnosis of isolated Horner confirmed by pharmacologic testing and/or documentation by neuro-ophthalmologists (SK and LBD) were included. The inclusion criteria also required neck computed tomography (CT) with contrast and/or CT angiography (CTA) imaging obtained within 6 months of the diagnosis. All studies were performed at the same institution. The exclusion criteria included the identification of any etiology of Horner syndrome on chart review or lack of imaging within the designated time frame.

A neuroradiologist (AS) with >20 years of experience, masked to the laterality of symptoms reviewed all CT scans, documenting the presence and severity of foraminal stenosis on each side at C7-T1, T1-2, and T2-3 levels on a 4-point Likert scale defined as follows: 0, none; 1, mild; 2, moderate; and 3, severe narrowing, with grades 1, 2, and 3 signifying <1/3rd, 1/3rd–2/3rd, and >2/3rd stenosis of the expected normal foraminal dimension. A cumulative foraminal stenosis score, representing the sum of stenosis grade at C7-T1, T1-2, and T2-3 levels was calculated for neural foramina ipsilateral and contralateral to the side of Horner syndrome. Group comparisons for ipsilateral and contralateral foraminal stenosis were done for each segmental level for maximum foraminal stenosis and cumulative foraminal stenosis scores using a *t*-test for paired data. A *p*-value of <0.05 was considered statistically significant. Descriptive statistics were used to summarize patient characteristics.

## Results

**Figure 1:**
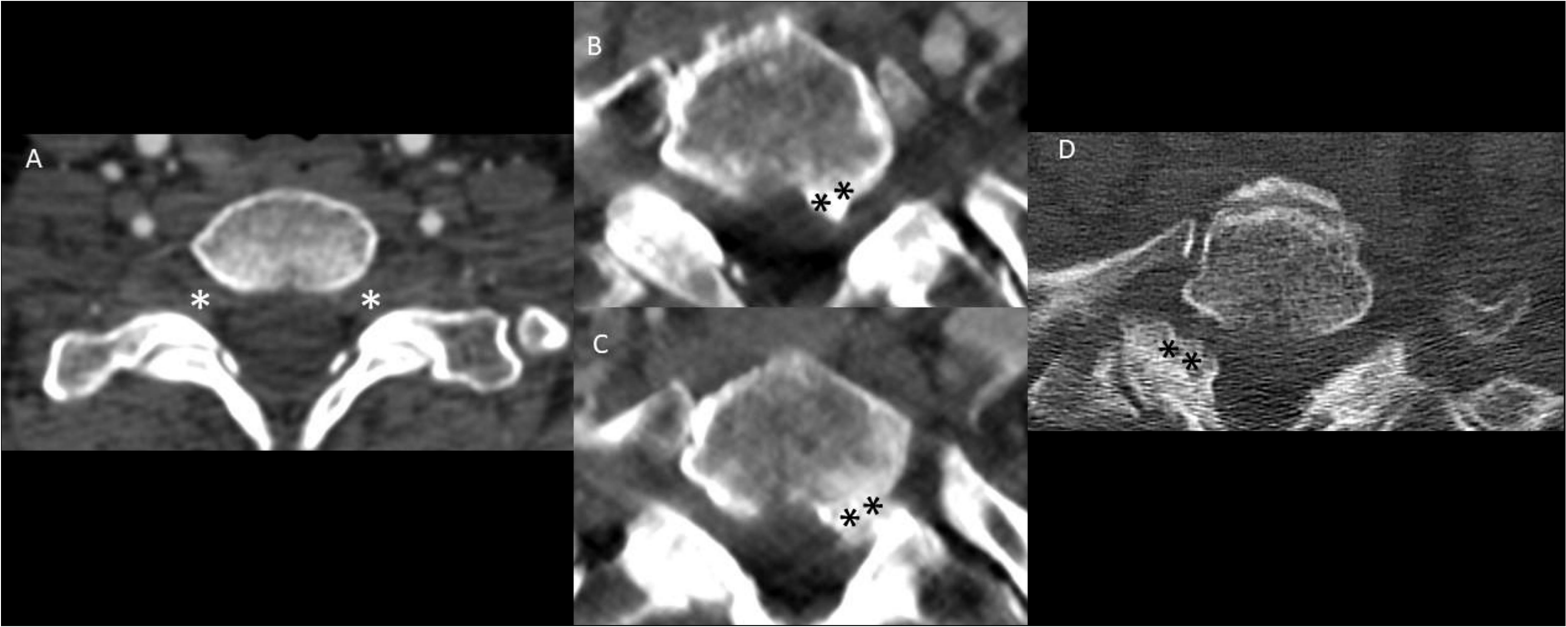
Spectrum of foraminal stenosis on computed tomography (CT) of the neck in Horner syndrome without any other identifiable cause. The majority of patients had patent bilateral neural foramina without any stenosis **(A)**. When present, foraminal stenosis was seen in both ipsilateral **(B, C)** and contralateral **(D)** to the side of Horner syndrome. Note the moderate asymmetric narrowing of the left T1-2 neural foramen **(B, C)** in a patient with left-sided Horner syndrome and the moderate asymmetric narrowing of the right T1-2 neural foramen **(D)** in a patient with left-sided Horner syndrome. “*” signifies the absence of foraminal stenosis at the C7-T1 level and “**” signifies hypertrophy.

**Figure 2:**
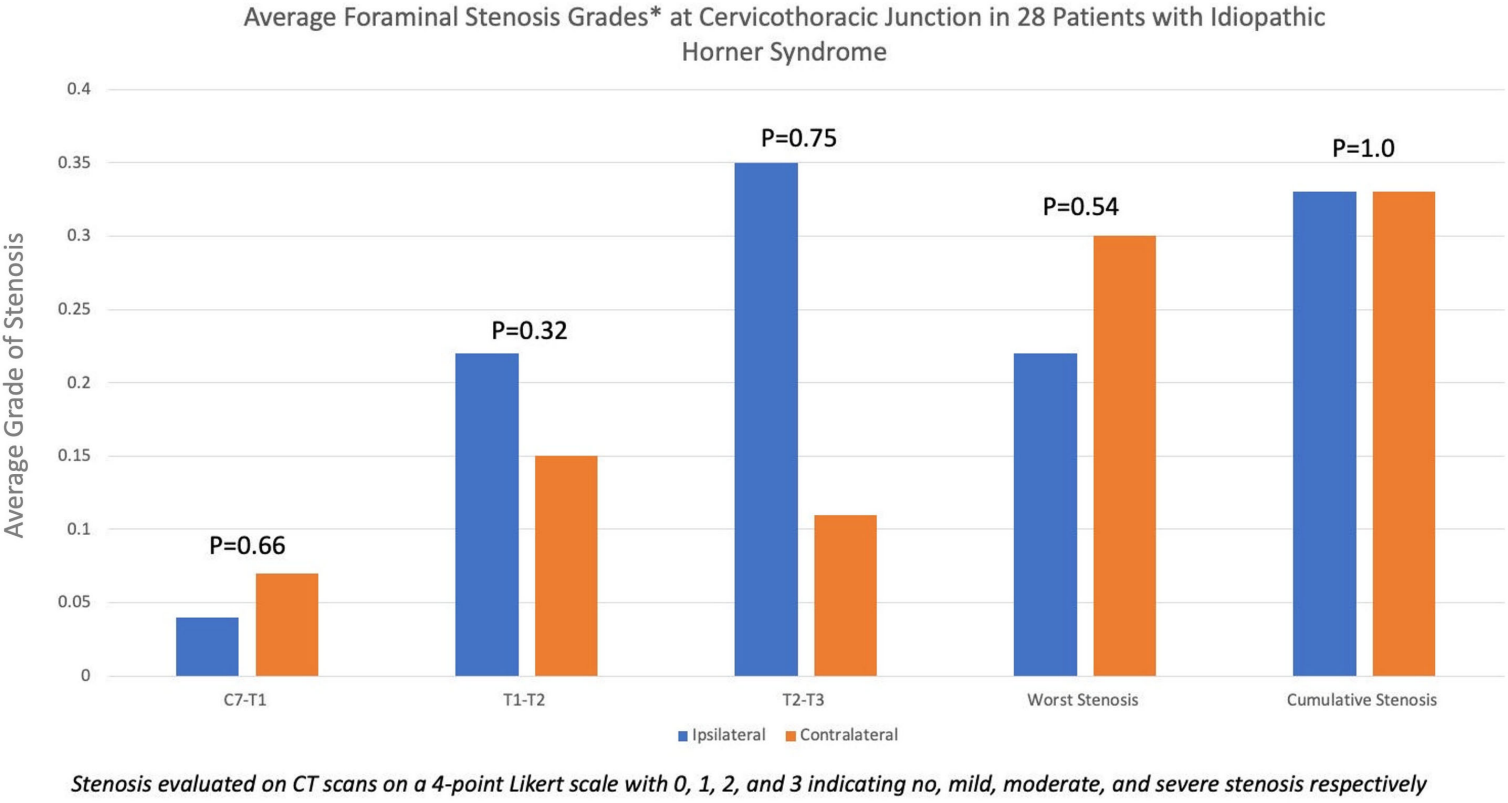
Bar diagram showing the average grade of foraminal stenosis between the C7 and T2 segments of the spine in patients with Horner syndrome, deemed to be idiopathic in nature. In general, average foraminal stenosis grades were low, indicating a low overall prevalence of foraminal stenosis at the cervicothoracic junction. The extent of stenosis was equivalent to ipsilateral and contralateral to the side of Horner syndrome.

Of the 336 patients seen at our institution between 2012 and 2022 with an ICD-9/10 diagnosis of Horner syndrome, the diagnosis was confirmed in 160 patients, of whom 41 were deemed to be idiopathic. In total, 28 of these patients for whom a CT scan was available within 6 months of diagnosis were included in the study. The average (± standard deviation) age of the patients qualifying for the study was 54.5 ± 18.7 years. A total of 18 were women and 10 were men. Foraminal stenosis was present in six (21.4%) patients, three (10.7%) of whom had bilateral stenosis. Other three (10.7%) patients had unilateral foraminal stenosis: one ipsilateral to the side of Horner syndrome in one and two contralateral to the side of Horner syndrome. Overall, stenosis was seen ipsilateral to the side of Horner syndrome in four (14.3%) patients and on the contralateral side in five (17.8%) patients (**Figure 1**). No significant difference in the extent of ipsilateral and contralateral foraminal stenosis was present at C7-T1 (*p* = 0.66), T1-2 (*p* = 0.32), or T2-3 (*p* = 0.75) levels. The average (± standard deviation) ipsilateral and contralateral cumulative foraminal stenosis scores were 0.33 ( ± 1.0) and 0.33 ( ± 1.1), respectively, with no significant difference (*p* = 1). The average (± standard deviation) maximum foraminal stenosis scores ipsilateral and contralateral to the side of Horner syndrome were 0.22 ( ± 0.59) and 0.30 (± 0.81), respectively, with no significant difference (*p* = 0.54) (**Figure 2**).

## Discussion

In various series, 23%–80% of all patients presenting with Horner syndrome end up being labeled as idiopathic without the establishment of an underlying cause. In our analysis, 25.6% (41/160) of all cases in which a diagnosis of Horner syndrome was confirmed were deemed idiopathic. The results of our study—showing both a low prevalence of foraminal stenosis at C7-T3 levels and an equivalent prevalence and severity of foraminal stenosis ipsilateral or contralateral to the symptomatic side—indicate that foraminal stenosis is unlikely to be a major causative etiology in such cases. Several case reports have previously described the development of Horner syndrome as a result of disc herniations at the cervicothoracic junction (7, 9–11). Even in our study, there were individual patients with moderately severe foraminal narrowing ipsilateral to the side of Horner syndrome. Accordingly, our results should not be taken as an argument against the possibility of foraminal narrowing leading to Horner syndrome in individual patients. Rather, our study suggests that such patients are likely to be rare. Despite these findings, there is no study to our knowledge that has analyzed this as a potential etiology of Horner syndrome, thus adding to the current literature and understanding of Horner syndrome.

The prevalence of disc herniations and other degenerative changes in the upper thoracic spine is very low, probably because of the stabilization provided by the rib cage. Our results demonstrate that this remains the case even in patients with Horner syndrome, which is deemed to be idiopathic. In addition to disc herniations, Horner syndrome has also been reported as a result of other spinal pathologies such as syringomyelia, intradural arachnoid cysts, spinal neoplasms, and epidural abscesses (12–17). Rather than focusing on the spinal canal, we explored foraminal narrowing as a possible cause of Horner syndrome because lesions affecting part of the oculosympathetic fibers within the spinal cord are likely to be associated with other signs of myelopathy and accordingly would be unlikely to remain otherwise asymptomatic. In contrast, we focused on foraminal stenosis resulting from degenerative changes in the intervertebral discs and/or facet joints, which are often found in asymptomatic populations and may often go unrecognized.

The main limitations of our study include the relatively small number of patients and the restriction of the analysis to CT scans rather than MRI. It is of note that at the authors’ institutions, CT/CTA of the head and neck is the imaging modality of choice for the investigation of isolated Horner; thus, the primary available imaging in this study was CT. While a larger number of patients might add statistical power, we think that our study—by showing a very low prevalence of foraminal stenosis in patients with Horner syndrome—is robust enough to convey that foraminal stenosis cannot be the likely explanation for a large number of patients with otherwise idiopathic Horner syndrome. While CT is excellent at delineating the bony changes resulting in foraminal narrowing, MRI is more useful for the delineation of disc herniations, and the inclusion of patients with both MRI CT would have provided a more comprehensive assessment of the spinal canal and the neural foramina, as MRI allows for direct imaging of the nerve roots. However, despite these limitations, we think that our methodology is robust enough to test our central hypothesis, adding novel and useful information to the existing knowledge about the possible pathogenesis of Horner syndrome.

## Conclusion

This study found a low prevalence of foraminal stenosis at C7-T3 segments in patients with idiopathic Horner syndrome. There was an equivalent prevalence and severity of foraminal stenosis ipsilateral and contralateral to the side of Horner syndrome. These results suggest that foraminal stenosis is unlikely to be a significant causative mechanism for Horner syndrome, which is otherwise deemed idiopathic.

## Data Availability

The raw data supporting the conclusions of this article will be made available by the authors, without undue reservation.
